# High-dose chemotherapy and autologous hematopoietic stem cell transplantation for progressive systemic sclerosis: a retrospective study of outcome and prognostic factors

**DOI:** 10.1007/s00432-024-05815-1

**Published:** 2024-06-08

**Authors:** Vanessa Pyka, Deepak B. Vangala, Thomas Mika, Alexander Kreuter, Laura Susok, Xenofon Baraliakos, Hannes Treiber, Roland Schroers, Verena Nilius-Eliliwi

**Affiliations:** 1 Department of Hematology and Oncology, Ruhr-University Bochum, Knappschaftskrankenhaus, In der Schornau 23-25, 44892 Bochum, Germany; 2 Department of Human Genetics, Ruhr-University Bochum, Bochum, Germany; 3 Department of Dermatology, Venerology and Allergology, HELIOS St. Elisabeth Klinik Oberhausen, University Witten-Herdecke, Oberhausen, Germany; 4 Department of Dermatology, Venerology and Allergology, Klinikum Dortmund, University Witten-Herdecke, Dortmund, Germany; 5 Department of Dermatology, Venerology and Allergology, Ruhr-University Bochum, Bochum, Germany; 6 Rheumazentrum Ruhrgebiet, Ruhr-University Bochum, Herne, Germany; 7 Department of Hematology and Oncology, Georg-August University, Göttingen, Germany

**Keywords:** Treatment-related mortality, Systemic sclerosis, Stem cell transplantation, Cell therapy, Hematopoietic cell transplantation-specific comorbidity index

## Abstract

**Purpose:**

Systemic sclerosis (SSc) is a rare autoimmune disease associated with high morbidity and mortality. SSc treatment is still challenging, and evidence is scarce. In the last decades high-dose chemotherapy and autologous stem cell transplantation (HD-ASCT) has proven to be effective. However, treatment related morbidity and mortality (TRM) are high. We conducted a retrospective, single-center analysis of SSc patients following HD-ASCT focusing on TRM and risk factors.

**Methods:**

32 patients who underwent HD-ASCT at our hospital between June 2000 and September 2020 were included. Clinical characteristics were evaluated based on chart review before and after HD-ASCT. Analyses focused on overall survival (OS), TRM, and response to HD-ASCT.

**Results:**

Median OS was 81 months (range 0–243). Within one year, 20 of 32 (76.9%) patients responded to HD-ASCT. Overall, 6 patients (18.8%) died in the context of HD-ASCT. Patients with subjective response to HD-ASCT (*p* = 0.024) and those with shorter time to platelet engraftment (*p* = 0.047) had significantly longer OS. Impaired renal function, age at HD-ASCT ≥ 55, disease duration < 12 months, high Hematopoietic cell transplantation-specific comorbidity index (HCT-CI) and Charlton Comorbidity Index (CCI) scores were associated with higher TRM. Patients receiving conditioning chemotherapy with thiotepa needed longer time for neutrophil (*p* = 0.035) and platelet engraftment (*p* = 0.021).

**Conclusion:**

This study confirms the efficacy of HD-ASCT for patients with SSc in a single center real-world setting. High TRM is still a challenge. However, TRM could be reduced by exclusion of high-risk patients and attention to prognostic parameters and scores as suggested in this study.

## Introduction

Systemic sclerosis (SSc) is a rare autoimmune disease. It is characterized by pathologic accumulation of extracellular matrix in connective tissue, most probably due to dysregulated and dysfunctional repair mechanisms. This leads to sclerosis of skin, vessels, and organs, especially including heart and lungs. Although an increasing number of immunosuppressive drugs have expanded the therapeutic options, SSc still has the highest mortality rate of all rheumatic diseases with estimated 10-year survival rates between 55 and 73% (Tyndall et al. 2010; Nikpour and Baron 2014; Elhai et al. 2017; Bergamasco et al. 2019).

Among immunosuppressive drugs, intravenous cyclophosphamide is a widely accepted first-line medication due to its positive effects on various organ involvements, especially for SSc-related lung disease (Tashkin et al. 2006, 2016). In recent years, the concept of high-dose chemotherapy followed by autologous stem cell transplantation (HD-ASCT) has become increasingly important. The simplified rationale behind this treatment is that high-dose chemotherapy depletes autoreactive immune cells, which are thought to be at least partially responsible for the pathogenesis of SSc, and thereby a kind of immunological reset is accomplished (Del Papa et al. 2018).

Three randomized controlled trials demonstrated the superiority of HD-ASCT over cyclophosphamide (ASTIS-Trial, ASSIST-Trial, SCOT-Trial (Burt et al. 2011; van Laar et al. 2014; Sullivan et al. 2018)). Compared to conventional cyclophosphamide protocols, HD-ASCT resulted in longer overall survival (OS), extended progression-free survival (PFS), and improved partial remission rates of organ involvement. Despite these positive effects of HD-ASCT, treatment-related mortality (TRM) and complication rates remain challenging (Henes et al. 2021). Studies have reported TRM rates mostly between 4 and 17% (Binks et al. 2001; Vonk et al. 2008; Burt et al. 2011, 2013; van Laar et al. 2014; Henes et al. 2014, 2021; Del Papa et al. 2018; Sullivan et al. 2018; van Bijnen et al. 2020; Henrique-Neto et al. 2021; Blank et al. 2022). In current studies, male sex and older age were associated with higher TRM and morbidity (van Bijnen et al. 2020; Henes et al. 2021; Spierings et al. 2022). Overall, treatment-related deaths are often due to cardiac complications (Burt et al. 2013; van Bijnen et al. 2020; Henes et al. 2021). To select patients who will probably benefit from the advantages of HD-ASCT, there is a need to identify factors predicting risks and long-term outcome after HD-ASCT. However, the predictive power of comorbidity indices such as the Charlson Comorbidity Index (CCI) and the Hematopoietic cell transplantation-specific comorbidity index (HCT-CI) on OS, PFS, or TRM is largely unexplored in the setting of SSc and HD-ASCT (Charlson et al. 1987; Sorror et al. 2005, 2014).

Furthermore, the optimal timepoint for HD-ASCT in the course of the disease is still unclear. Considering the risks and potential long-term side effects, HD-ASCT is often performed in rather advanced SSc stages. However, this potentially results in increased complication rates, as SSc-associated organ damage reduces the functional reserve during treatment complications (Vonk et al. 2008; van Laar et al. 2014; Del Papa et al. 2018; van Bijnen et al. 2020).

Regarding outcome measures for HD-ASCT in SSc, research has previously focused on the relevance of patients' general condition and subjective perception. Evaluation with standardized health-related quality of life questionnaires shows an improvement in health-related quality of life in patients treated with HD-ASCT in comparison to conventional therapy (Maltez et al. 2021).

In this retrospective study, we present real-world data of 31 SSc patients treated with HD-ASCT at a tertiary referral center since 2000. Detailed analyses of outcome parameters including TRM, procedure related side effects, OS, and potential risk factors for HD-ASCT are discussed.

## Methods

### Study design and population

This retrospective, single-center study included 31 SSc patients, who received HD-ASCT at the University Hospital Knappschaftskrankenhaus Bochum, Germany, between June 2000 and September 2020. A total of 31 patients were included, of which one patient had received HD-ASCT twice. For the sake of simplicity, we will therefore refer to 32 performed HD-ASCTs in the further course. Three patients had also been included in the ASTIS trial and some of the patients participated in the DNSS study (van Laar et al. 2014; Blank et al. 2022). The local ethics committee of the Ruhr-University Bochum approved this study (No. 21–7212-BR).

### Treatments

All patients received cytotoxic stem cell mobilization with cyclophosphamide (2 g/m^2^) and G-CSF (10 µg/kg per day)). In 16 patients treated between 2000 and 2016, conditioning high-dose chemotherapy consisted of cyclophosphamide (50 mg/kg, days -7 to -3) and anti-thymocyte-globulin (ATG; 2.5 mg/kg, days -5 to -2) (CyATG) (van Laar et al. 2014). In one of these patients, the dose of cyclophosphamide was reduced to 75% considering cardiac disease involvement. One patient was treated with fludarabine, busulfan, and ATG. For the 15 patients who were treated between 2017 and 2020 conditioning chemotherapy comprised thiotepa (5 mg/kg, twice on day -7) combined with cyclophosphamide (50 mg/kg, days -4 and -3) and ATG (10 mg/kg, days -5 to -2) (TrCyATG) (Henes et al. 2014). One of these 15 patients received melphalan (100 mg/m^2^ on day -2) instead of cyclophosphamide because of cardiac SSc involvement.

In all protocols, cryopreserved autologous hematopoietic stem cells were reinfused on day 0. To avoid inflammatory rebound during hematopoietic reconstitution, patients did not receive G-CSF post HD-ASCT. Patients treated until 2014 received separated CD34 + stem cells (*n* = 12, 37.5%). Since 2015 cell selection for CD34 positivity was no longer performed (*n* = 20, 62.5%). Median dose of reinfused CD34 + stem cells in patients was 6.4 × 10^6^ (range 2.2–12.07 × 10^6^).

### Data collection

Patient data including several parameters for systemic organ involvement before and after HD-ASCT was collected based on chart reviews. CCI and HCT-CI were calculated for each patient at the timepoint of HD-ASCT. Disease duration was defined as time between the patient's symptom onset or initial diagnosis and HD-ASCT. Skin involvement was present if patients had cutaneous manifestations that could be attributed to SSc or had a pathological value in the modified Rodnan skin score (mRSS). We considered pulmonary involvement of SSc according to imaging if the examining radiologist detected typical SSc related changes on chest X-ray and HR-CT. Functional lung parameters included forced vital capacity (FVC) and diffusing capacity of the lungs for carbon monoxide (DLCO). The presence of pulmonary arterial hypertension was defined as a mean pulmonary arterial pressure greater than 25 mmHg measured via right heart catheterization or estimated either with cardiac MRI or echocardiography.

Cardiac involvement was assessed based on echocardiography and cardiac MRI (Candell-Riera et al. 1996; Ferri et al. 2003; Bezante et al. 2007; Kahan et al. 2009). Patients were classified with gastrointestinal SSc involvement in case of typical symptoms (e.g. dysphagia, reflux/regurgitation), pathological findings in barium swallow tests, or typical endoscopic signs (e.g. reflux esophagitis).

Joint involvement was diagnosed based on either imaging findings (skeletal scintigram, X-ray, and/or MRI) or if the patient suffered from joint complaints such as pain, stiffness, or swellings due to synovitis, which could be plausibly attributed to SSc in the overall view of clinical symptoms and findings.

### Study parameters

Study parameters were overall survival (OS) after HD-ASCT and treatment-related mortality (TRM).

OS was defined as the time between transplantation and patient death as well as last contact or cut-off. Death was classified as treatment related if the patient died within 100 days of transplantation and/or the procedure of HD-ASCT was the major causal influencing factor.

In addition, the patients’ subjective responses to HD-ASCT, cutaneous improvement, dynamic development of DLCO and FVC, and overall response (OR) within one year post HD-ASCT were evaluated.

Under the broad parameter "cutaneous response" a response of the mRSS and improvement of the SSc-typical cutaneous manifestations are subsumed. In case of at least 25% mRSS reduction compared to baseline before HD-ASCT, improvement of mRSS was documented.

Lung function was evaluated on basis of DLCO and FVC. Changes in both parameters were categorized as following: “no change” for 0–9%, “improvement” for increases of ≥ 10%, or “deterioration” for decreases of ≥ 10% from baseline before HD-ASCT. A positive subjective response was determined if the individual patient reported a clear improvement of clinical symptoms compared to the status prior to HD-ASCT. OR denotes the presence of any response in at least one category listed above.

### Statistical analysis

Data was analyzed with central tendency measures, boxplots, and frequencies. Due to small numbers, all continuous variables were tested for normal distribution using the Shapiro–Wilk test. The probabilities of OS were calculated using the Kaplan–Meier method. Two formed groups of patients on different independent variables were compared for the probability of OS after HD-ASCT using log-rank tests. An association between continuous/ordinal variables and OS was evaluated using Pearson’s or Spearmann’s correlation, depending on the scale level and the distribution of variable’s values. Association between TRM and categorical variables was examined using the chi-square test, unpaired T test for continuously normally distributed variables, and Mann–Whitney U test for continuously non-normally distributed variables respectively.

Parameters before and after HD-ASCT were compared using paired-samples T tests for normally distributed variables and Mann–Whitney U tests for non-normally distributed variables. For multivariate analysis we applied Cox regression analysis. Statistical significance was defined as *p* < 0.05. All analyses were performed using IBM SPSS Statistics 28. Figures were generated using R.

## Results

### Patient characteristics

Median follow-up after HD-ASCT was 29.5 months (0–243 months). A total of 20 patients were female (62.5%), and 12 patients male (37.5%). Median time between initial diagnosis and HD-ASCT was 44.5 months (range 2–328 months). Median time between onset of first symptoms and HD-ASCT was 54 months (range 2–405 months). Median age at HD-ASCT was 51.5 years (range 25–62 years). Of 32 patients, 13 (40.6%) were active smokers.

At the time of HD-ASCT, patients had a median CCI of 2 (range 1–9), HCT-CI of 6 (range 4–10), and age-adjusted HCT-CI of 7 (range 4–11), respectively. Skin involvement was detectable in all patients with a median mRSS of 23 (range 9–48). Cardiac involvement as based on imaging was detected in 22 of 32 (68.8%) patients. According to radiological findings, lungs were affected in 27 of 32 (84.4%) patients. Clinically relevant pulmonary arterial hypertension was detected in 11 of 23 (47.8%) patients. Median diffusion capacity (DLCO) was 49.0% (range 21–64%) and median forced vital capacity (FVC) was 70.5% (range 33–97%) of the respective target values. Gastrointestinal and joint involvement were found in 26 of 32 (81.3%) and 26 of 30 (86.7%) patients, respectively. Anti-topoisomerase-I antibodies were detected by standard ELISA in 17 of 25 (68%) patients. All relevant patient characteristics are shown in Table 1.

**Table 1:** Baseline characteristics before HD-ASCT

Parameters	
Patients, no.	31
HD-ASCT procedures, no.	32
Disease duration from SSc diagnosis, median (range), months	44.5 (2–328)
Age at HD-ASCT, median (range), years	51.5 (25–62)
Sex, female, no. (%)	20 (62.5)
Active Smokers, no. (%)	13 (40.6)
Charlson Comorbidity Index, median (range)	2 (1–9)
HCT-CI, median (range)	6 (4–10)
HCT-CI (age adjusted), median (range)	7 (4–11)
Modified Rodnan Skin Score, median (range)*	23 (9–48)
Lung involvement	
DLCO, % from predicted value, median (range)*	49 (21–64)
FVC, % from predicted value, median (range)*	70.5 (33–97)
Lung involvement according to imaging, no. (%)	27 (84.4)
Considered by chest X-ray, no. (%)	4 (14.8^#^)
Considered by HR-CT, no. (%)	23 (85.2^#^)
Pulmonal arterial hypertension (mPAP > 25 mmHg), no. (%)*	11 (47.8)
Estimated by TTE or Cardiac-MRT, no. (%)	7 (87.5^#^)
Considered by right heart catheterization, no. (%)	1 (12.5^#^)
Cardiac involvement	
LVEF < 50%, no. (%)*	5 (17.2)
Cardiac involvement according to imaging, no. (%)	22 (68.8)
Considered by echocardiography	9 (40.9^#^)
Considered by Cardiac-MRT	13 (51.1^#^)
Kidney involvement	
GFR according CDK-EPI equation, median (range), ml/min*	97 (31–144)
Proteinuria, no. (%)*	5 (16.1)
Gastrointestinal involvement, no. (%)*	26 (81.3)
Considered by clinical symptoms	5 (19.2^#^)
Considered by barium swallow or endoscopy	21 (80.7^#^)
Joint involvement, no. (%)*	26 (86.7)
Considered by clinical symptoms	15 (57.7)
Considered by imaging (skeletal scintigram, X-ray, MRI)	11 (42.3)
SSc-Antibodies	
Anti-nuclear antibody positive, no. (%)*	18 (94.6)
Anti-topoisomerase-I (Scl-70) positive, no. (%)*	17 (68)
Previous immunosuppressive SSc medication, no. (%)	
Cyclophosphamide	18 (56.3)
Methotrexate	16 (50.0)
Mycophenolate mofetil	1 (3.1)
Prednisone	15 (46.9)
Azathioprine	6 (18.8)
Cyclosporin	2 (6.3)
Conditioning chemotherapy protocol, no. (%)	
CyATG protocol	16 (50)
TrCyATG protocol	15 (46.9)

^*^Incomplete data, % from patients with available data

^#^Percentages based on the number of HD-ASCT procedures of the heading category as reference population

*HD-ASCT* High dose chemotherapy with autologous stem cell transplantation, *HCT-CI* hematopoietic cell transplantation specific comorbidity index, *DLCO* diffusion capacity of lung for carbon monoxide, *FVC* forced vital capacity, *HR-CT* high-resolution computer tomography, *TTE* transthoracic echocardiogram, *LVEF* left ventricular ejection fraction, *GFR* glomerular filtration rate, *CDK-Epi equation* chronic kidney disease epidemiology collaboration equation, *SSc* systemic sclerosis

### Survival analyses

**Fig. 1:**
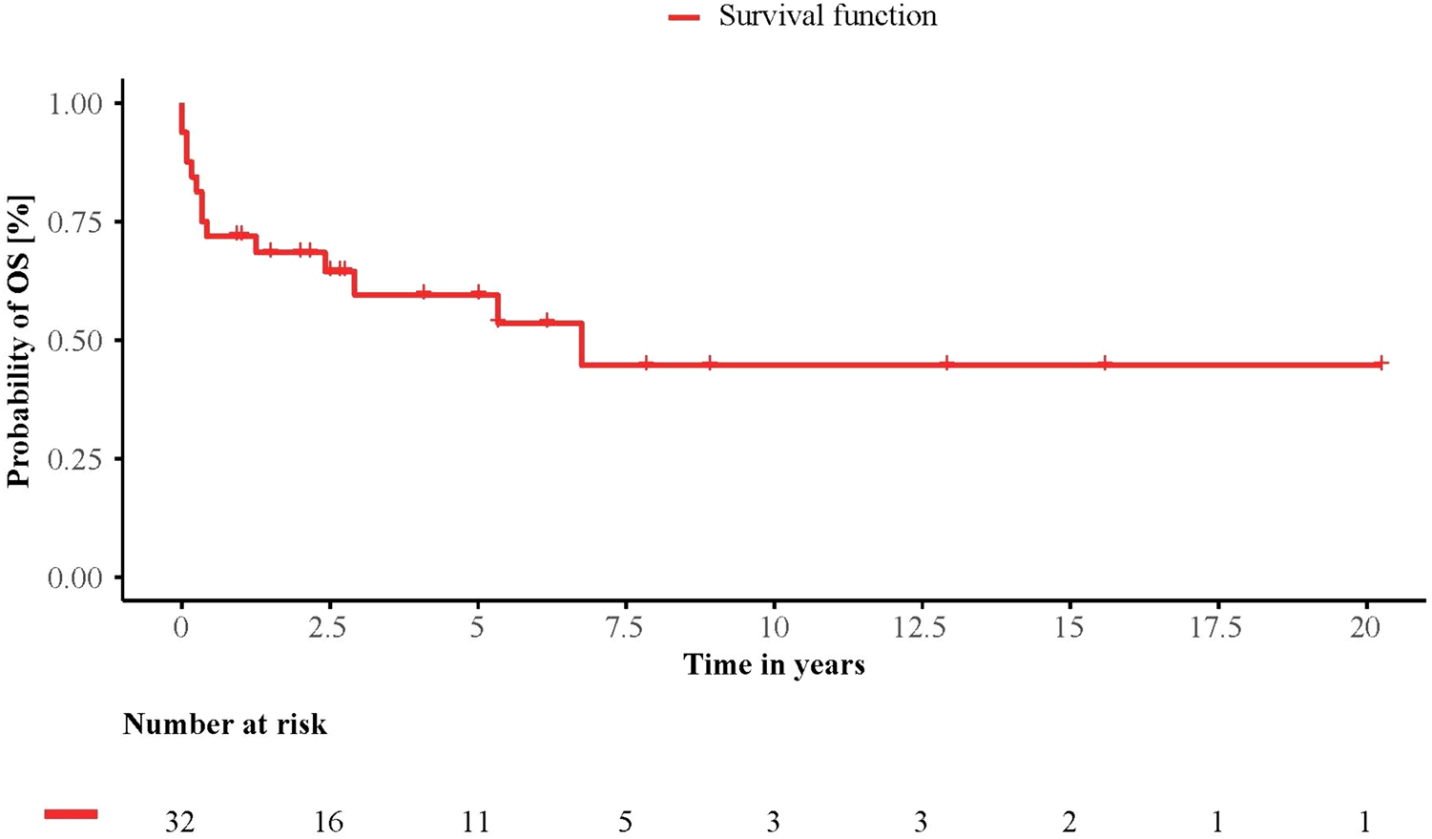
Overall survival. Kaplan–Meier curve showing survival after high-dose chemotherapy and autologous stem cell transplantation (day 0) for all patients (*n* = 32)

Median OS of the whole cohort was 81 months (0–243). OS rates at 1 year and 5 years were 71.9% and 59.5%, respectively (Fig. 1). In total, 14 (43.8%) patients died at the time of analysis. Overall, 6 patients (18.8%) died treatment-related post HD-ASCT, and 8 (25%) patients due to SSc progression.

**Fig. 2:**
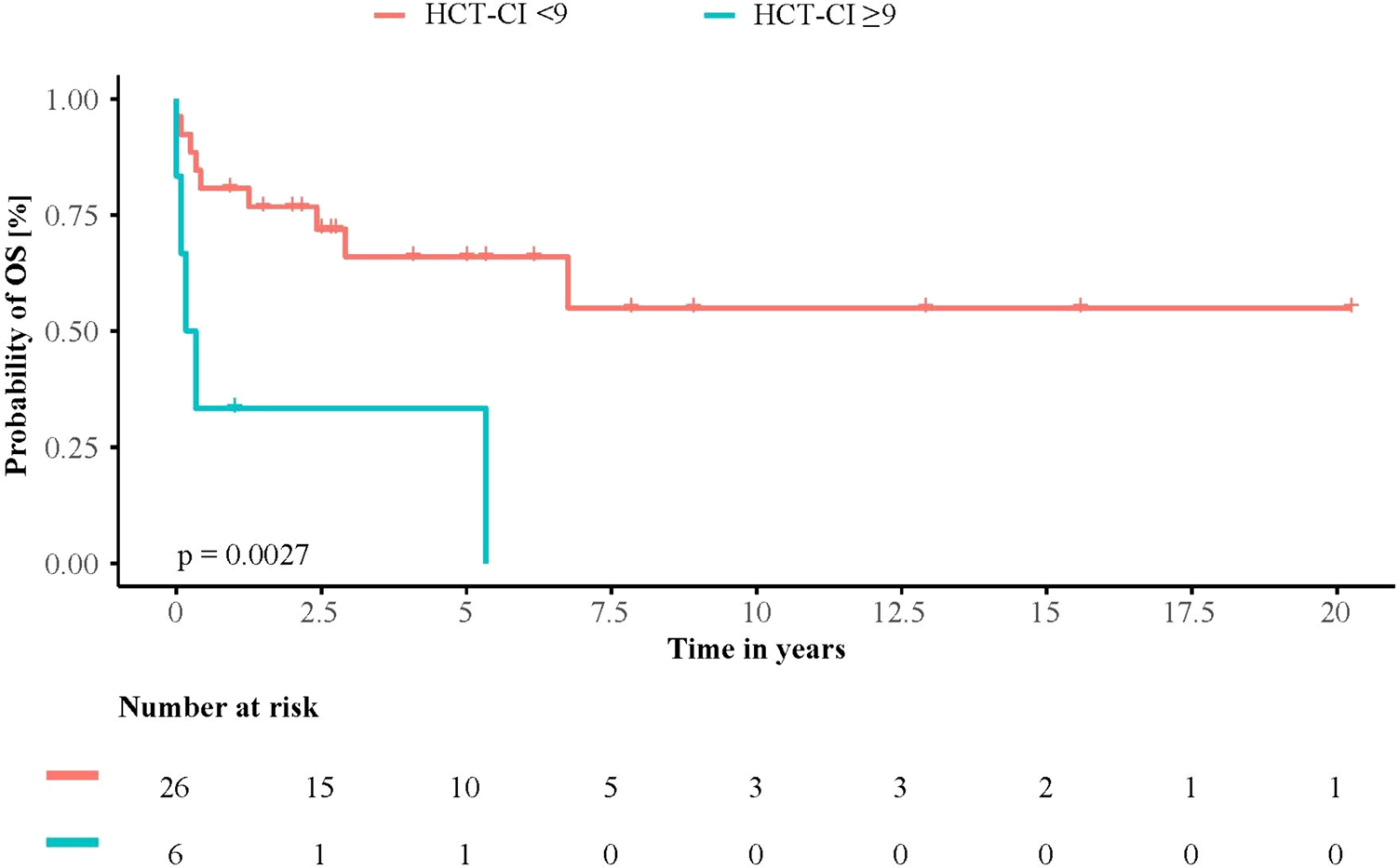
Overall survival (OS) by high and low Hematopoietic cell transplantation specific comorbidity index (HCT-CI). Kaplan–Meier curve showing survival of patients with high (≥ 9, *n* = 6, 18.7%) and low HCT-CI (< 9, *n* = 26, 81.3%). Patients with low HCT-CI (< 9) had significantly longer OS than patients with high HCT-CI (≥ 9) (*p* = 0.003)

Detailed analysis of patient factors associated with OS showed, patients without gastrointestinal involvement (*n* = 6; 19.4%; log-rank p = 0.036), with lower conventional HCT-CI (< 9, *n* = 26, 81.3%; log-rank *p* = 0.003, Fig. 2) as well as lower age-adjusted HCT-CI (< 10, *n* = 28, 87.5%; log-rank *p* = 0.008) had significantly longer OS.

**Fig. 3:**
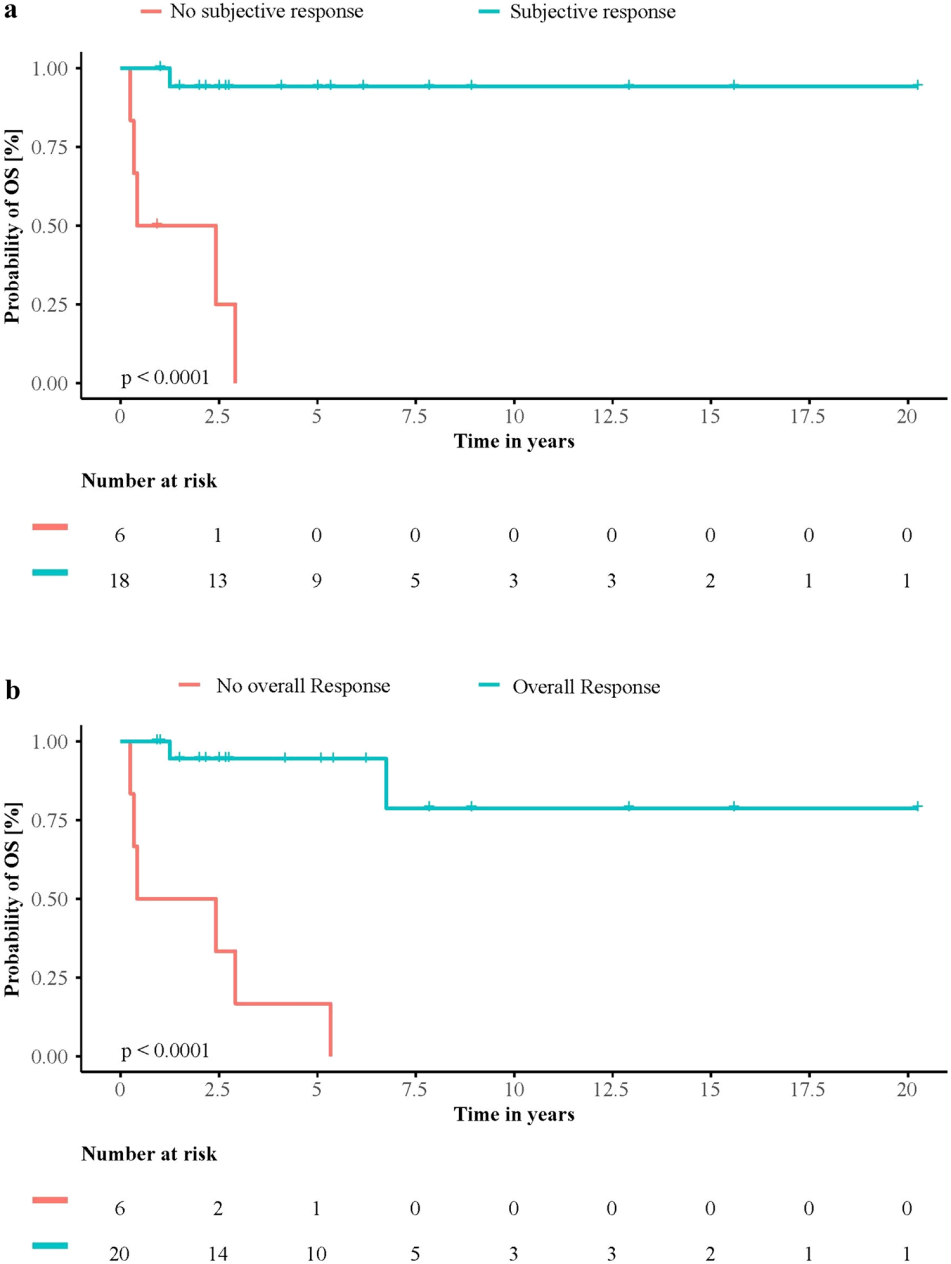
**a** Overall survival by subjective response. Kaplan–Meier curve showing survival of patients with (*n* = 18, 75%) or without (*n* = 6, 25%) subjective response to HD-ASCT. Patients with subjective response had significantly longer OS than patients, who showed no subjective response (*p* < 0.001). **b** Overall survival by overall response (OR). Kaplan–Meier curve showing survival of patients with (*n* = 20, 76.9%) or without (*n* = 6, 23.1%) clinical response to HD-ASCT in at least one category (overall response; OR). Patients with OR had significantly longer OS than patients without (*p* < 0.001)

Patients reporting a subjective response after HD-ASCT had a highly significant longer OS as compared to those not reporting a subjective response (*n* = 18, 75.0%; log-rank *p* < 0.001; Fig. 3a). Likewise, patients with a present overall response (OR) had a significantly longer OS as compared to patients with no OR (*n* = 20, 76.9%; *p* < 0.001, Fig. 3b). However, response in one of the other categories did not correlate with OS.

Interestingly, there was a significant negative association between time to platelet engraftment and OS (*r* = −0.431; *p* = 0.025).

Female patients (*n* = 20, 62.5%; log-rank *p* = 0.097), patients with lower CCI (< 4, *n* = 24, 75%; log-rank *p* = 0.148), BMI in normal or above-average range (≥ 18, *n* = 30, 93.8%; log-rank *p* = 0.213), and a lower mRSS (< 25, *n* = 11, 52.4%; log-rank *p* = 0.166) showed a trend to longer OS when analyzing the Kaplan–Meier curves. Pulmonal (DLCO value < 45%, *n* = 11, 47.8%; log-rank *p* = 0.158) and cardiac (*n* = 22, 68.8%; log-rank *p* = 0.083) involvement were associated with a trend to shorter OS. However, these analyses did not reach statistical significance (log-rank *p* ≥ 0.05).

Differences in glomerular filtrations rate (GFR; < 90, *n* = 12, 38.7%; log-rank *p* = 0.165) and joint involvement (*n* = 26, 86.7%; log-rank *p* = 0.784) were not associated with OS. Moreover, patients with either short (< 36 months, *n* = 14, 43.8%) or long disease duration (≥ 36 months, *n* = 18, 56.8%) did not differ in OS (log-rank *p* = 0.638). Similarly, there was no significant difference in the OS of patients treated with the two different chemotherapy regimens, CyATG (*n* = 16, 50%) or TrCyATG (*n* = 15, 46.88%; log-rank *p* = 0.960) Likewise, no difference in OS was detected for patients receiving grafts with selected CD34 + stem cells (*n* = 12, 37.5%) and patients where selection for CD34 + stem cells was not performed (*n* = 20, 62.5%; log-rank *p* = 0.948).

### Treatment response to HD-ASCT

20 of 26 (76.9%) patients, that survived one year and longer after HD-ASCT developed a clinical response in at least one category (OR (overall response): subjective response, cutaneous response, dynamic development of DLCO and FVC). As mentioned above, these patients (*n* = 20, 76.9%) had significantly longer OS as compared to patients who did not respond in any category (*n* = 6, 23.1%; log-rank *p* < 0.001, Fig. 3b). In fact, response in the first year after HD-ASCT was the strongest parameter correlating with longer OS. Therefore, these patients were subsequently characterized in more detail. They showed a significantly higher FVC (47.00%, SD = 12.12 vs. 69.40%, SD = 13.08; *t*(16) = −2.73, *p* = 0.015) and more often a DLCO value above 30% (*n* = 13 vs. *n* = 1; Chi^2^(1) = 6.19, *p* = 0.013). Moreover, patients with a BMI ≥ 18 (*n* = 24, 92.3%) were more likely to show a response to HD-ASCT than patients with a BMI < 18 (*n* = 2, 7.7%; Chi^2^(1) = 7.22, *p* = 0.007).

Overall, 16 of 22 (72.7%) patients had cutaneous improvement. More precisely, 3 of 6 (50%) patients showed a response to HD-ASCT in mRSS and 14 of 21 (66.7%) patients in clinical cutaneous SSc manifestations. There was no significant worsening of mRSS comparing values before and after HD-ASCT (21.60, SD = 7.40 vs. 21.20, SD = 7.86; *t*(4) = 0.14, *p* = 0.898).

Regarding lung function, 15 of 19 (78.9%) patients showed improvement or stabilization in DLCO values and 16 of 17 (94.1%) patients in FVC values when timepoints before and after HD-ASCT were compared.

Mean BMI was 23.78 before transplantation and 23.42 after HD-ASCT, showing no statistically significant decrease (23.78, SD = 4.01 vs. 23.42, SD = 3.48; *t*(17) = 0.67, *p* = 0.509).

### HD-ASCT, Safety, TRM

Median time to neutrophil and platelet engraftment were 11 (range 8–19) and 10 (range 7–18) days, respectively. Overall, TRM was 18.8% with 6 patients dying due to side-effects of HD-ASCT. Four of 6 (66.7%) patients died due to infectious complications, either due to pneumonia or neutropenic sepsis. One of these 6 (16.7%) patients died due to cardiac failure. Another patient died 125 days after HD-ASCT with multiple organ failure following prolonged intensive care unit treatment.

To further understand the occurrence of TRM we analyzed potential patient-related risk factors associated with TRM. Impaired renal function (GFR < 90 ml/min, *n* = 12, 38.7%; Chi^2^(1) = 6.24, *p* = 0.012), age at HD-ASCT above 55 years (*n* = 12, 37.5%; Chi^2^(1) = 6.62, *p* = 0.010), CCI ≥ 4 (*n* = 8, 25%; Chi^2^(1) = 6.84, *p* = 0.009), high conventional HCT-CI (≥ 9, *n* = 26, 81.3%; Chi^2^(1) = 11.13, *p* < 0.001), as well as age-adapted HCT-CI (≥ 10, *n* = 28, 87.5%; Chi^2^(1) = 9.5, *p* = 0.002) and disease duration since SSc diagnosis < 12 months (*n* = 6, 18.7%; Chi^2^(1) = 4.73, *p* = 0.030) were significantly associated with an increased risk of death following HD-ASCT. In detail, patients with disease duration < 12 months had an average eightfold (OR 7.67, 95%-CI 1.04–56.77) and patients with GFR < 90 ml/min a 12-fold increased risk for TRM (OR 12.86, 95%-CI 1.27–130.54), respectively. Patients older than 55 years had a 14-fold increased risk (OR 13.57, 95%-CI 1.34–137.45), whereas patients with CCI ≥ 4 had an 11-fold increased risk of therapy-associated death (OR 11.00, 95%-CI 1.48–81.61). Furthermore, patients with a higher conventional HCT-CI (≥ 9; OR 24.00, 95%-CI 2.59–222.65) and age-adapted HCT-CI (≥ 10; OR 25.00, 95%-CI 1.93–323.55) had a 24- and 25-fold risk of TRM respectively (Table 2).

**Table 2:** Odds Ratios (OR) for treatment-related mortality (TRM)

**Factors**	
HCT-CI (age adjusted) ≥ 10	OR 25.00 (95%-CI 1.932–323.553)
HCT-CI ≥ 9	OR 24.00 (95%-CI 2.587–222.646)
Age at transplantation ≥ 55 years	OR 13.57 (95%-CI 1.340–137.454)
GFR < 90 ml/min	OR 12.86 (95%-CI 1.266–130.535)
Charlson Comorbidity Index ≥ 4	OR 11.00 (95%-CI 1.483–81.606)
Disease duration from SSc diagnosis < 12 months	OR 7.67 (95%-CI 1.035–56.770)

OR for TRM for all factors significant in chi-square-test, which were associated with a higher prevalence of TRM. Shown in each case is the group of HD-ASCT procedures that included patients at higher risk for TRM. HD-ASCT procedures including patients with an high age-adjusted hematopoetic cell transplantation (HCT)-Comorbidity Index ≥10 (*n*=4, 12.5%) had a 25-fold increased risk for TRM (Chi^2^(1)=9.5, *p*=0.002). Similarly 24-fold increased risk for TRM was noticed for patients with a high conventional HCT-Comorbidity Index ≥9 (*n*=6, 18.8%) (Chi^2^(1)=11.13, *p*<0.001)

In addition, patients with cardiac involvement (*n* = 22, 68.8%; Chi^2^(1) = 3.36, *p* = 0.067), DLCO < 35% (*n* = 7, 30.4%; Chi^2^(1) = 2.12, *p* = 0.146), less prior immunosuppressive therapy (< 4 previous immunosuppressive medications; *n* = 25, 78.1%; Chi^2^(1) = 2.09, *p* = 0.150), joint involvement (*n* = 26, 86.7%; Chi^2^(1) = 1.15, *p* = 0.183) and gastrointestinal involvement (*n* = 25, 80.6%; Chi^2^(1) = 1.43, *p* = 0.232) also showed a tendency for increased mortality risk post HD-ASCT.

Interestingly, patients treated with the CyATG protocol (*n* = 16, 50.0%) or with the TrCyATG protocol (*n* = 15, 46.9%) showed no difference in risk for TRM (Chi^2^(1) = 0.24, *p* = 0.877). Similarly, TRM in patients receiving grafts with selected CD34 + cells did not differ from patients receiving unselected cells (Chi^2^(1) = 0.731, *p* = 0.393). Nevertheless, patients treated with TrCyATG had a longer time for neutrophil (16.21 days vs. 10.04 days; *U* = 116.50, *p* = 0.035) and platelet engraftment (12.58 days vs. 10.38 days; *T* = −2.47, df = 23, *p* = 0.021).

### Multivariate analysis

Multivariate Cox regression analysis was performed with the variables subjective response, gastrointestinal involvement, time to platelet engraftment and HCT-CI for OS. In multivariate analysis patients with subjective response (*p* = 0.024) or shorter time to platelet engraftment (*p* = 0.047) had a significantly higher OS. The presence of a subjective response within one year reduced the risk of death by more than 98% (HR: 0.016, 95%-CI 0.0–0.59). For every extra day to platelet engraftment, the risk of death increased approximately 2.3-fold (HR: 2.26, 95%-CI: 1.01–5.04). Gastrointestinal involvement (*p* = 0.973) and HCT-CI (*p* = 0.985) did not reach significance in multivariate analyses.

## Discussion

SSc is a systemic autoimmune disease, for which HD-ASCT has proven to be superior to standard cyclophosphamide in several prospective clinical trials. Up to now only few studies exist, analyzing the risks and benefits of this infrequently used therapy in a real-world setting. Therefore, with this retrospective, single-center study we aimed to analyze the effectivity and safety of HD-ASCT in a real-world setting with a special focus on TRM and factors associated with TRM.

Notably, in our cohort 1- and 5- year OS rates were lower than those of other studies with 71.9% and 59.5%, respectively (Fig. 1). In the literature, for example 1-year survival rates are found in the range of 80–95% (van Laar et al. 2014; Henes et al. 2021). This was driven by an increased TRM of 18.8% in our cohort, as compared to other studies with approximately 0–17%, but mostly in the range of 5–10% (Binks et al. 2001; Vonk et al. 2008; Burt et al. 2011, 2013; van Laar et al. 2014; Henes et al. 2014, 2021; Del Papa et al. 2018; Sullivan et al. 2018; van Bijnen et al. 2020; Henrique-Neto et al. 2021; Blank et al. 2022). However, due to the lack of treatment alternatives the potentially life-saving HD-ASCT was offered to some patients in our center, which would have been excluded in clinical trials. To strengthen this assumption about our cohort, we followed the ASTIS study and applied its exclusion criteria to our patient population. According to the ASTIS study, patients with severe organ involvement or with severe comorbidities were excluded (van Laar et al. 2014). Translating these criteria to our patient cohort, we defined a group of patients who had a mPAP > 50 mmHg, CCI ≥ 4, DLCO < 25%, GFR < 45 ml/min, FVC < 40%, oxygen mandatory pulmonary insufficiency, or severe comorbidities such as COPD or myocardial infarction. Those 16 patients would have been excluded following ASTIS criteria. For those severely ill patients, median OS was 29 months whereas median was not reached for the fitter patients. Among these, only one treatment-related death occurred. Accordingly, TRM was reduced from 18.8% (*n* = 6) to 6.3% (*n* = 1), which is in the range of the prospective studies mentioned above.

Our retrospective observations are supported by another study by Binks et al. including a comparable patient population which had similar survival rates (1-year OS 73%) and TRM (NRM 17%) (Binks et al. 2001). Not all of our patients who were excluded according to ASTIS criteria or with comorbidity scores died related to HD-ASCT. For example, one patient with a CCI ≥ 4 was still alive 60 months after HD-ASCT. Another patient had exhibited an HCT-CI > 9, however, he survived HD-ASCT and had a favorable clinical response. Accordingly, these cases illustrate that even high-risk patients potentially benefit from HD-ASCT in advanced stages, which is line with the findings of a recently published study (Spierings et al. 2022).

A major purpose of our work was to identify factors potentially associated with TRM, in order to provide a rationale for selecting patients likely benefitting from HD-ASCT (Table 2). That is why different scores, such as the CCI and its further development the HCT-CI have been established in the last years. The HCT-CI was originally developed in the setting of allogeneic stem cell transplantation. Its validity in relation to autologous stem cell transplantation is still debated and its predictive power for TRM especially in systemic sclerosis has not yet been clarified (Saad et al. 2014; Sorror et al. 2015; Berro et al. 2017; van Bijnen et al. 2020; Barth et al. 2022). Here, we demonstrated that patients with a high CCI and especially high HCT-CI were at significantly increased risk for TRM (OR 11, OR 24, respectively (Charlson et al. 1987; Sorror et al. 2005; Sorror et al. 2014)). Therefore, calculating those scores before HD-ASCT in SSc patients seems to be a reasonable tool to predict survival and could therefore be used to facilitate decisions for intensive therapies.

Furthermore, low GFR turned out to be significantly associated with TRM in our study cohort. It is debatable whether it is nevertheless useful to perform HD-ASCT in patients with severe renal involvement, as they have a very poor prognosis with a one-year survival rate of only 70% (Guillevin et al. 2012). In the present study, patients with impaired renal function (GFR < 90 ml/min) showed a one-year survival rate of 58%. No other organ involvement is associated with survival rates as low as scleroderma renal crisis. Therefore, HD-ASCT does harbor a high risk for TRM in these patients; however, the partial remission of the clinical picture due to transplantation may also prevent a future scleroderma renal crisis and terminal renal insufficiency in these patients as well as prolong survival in the long term.

Another important and still unanswered question is when to perform HD-ASCT during the course of disease. To address the question of HD-ASCT, we analyzed the time between symptom onset as well as initial diagnosis and transplantation in terms of OS and TRM. It could be shown that a particularly short period between SSc diagnosis and HD-ASCT (< 12 months) was associated with a higher risk of TRM. This can most likely be explained by the fact that a very short period between diagnosis and HD-ASCT is indicative for a very aggressive disease course and therefore leading to a worse outcome. Nevertheless, a better physical condition with less organ involvement improves patients´ outcome. Therefore, it could be important to have a close monitoring of the patients´ organ functions to ensure that the right timepoint for HD-ASCT is not missed. This is in line with the analyses of the recently published, large DNSS study that showed that patients with a disease duration of more than 3 years do not necessarily have a worse outcome after HD-ASCT (Blanks et al. 2022).

In this study, 75% of patients showed a therapeutic response to HD-ASCT within one year. Furthermore, there was no significant deterioration in the various organ parameters in the study cohort after HD-ASCT. In line with other studies, one can conclude that HD-ASCT has great therapeutic benefit for patients with SSc, which also holds true for patients in the real-world setting, even if the definition of response differs between studies (Vonk et al. 2008; Burt et al. 2011; Henes et al. 2021). Nevertheless 25% of patients do not respond and the risk of TRM is still eminent. Therefore, it is important to know, which groups of patients are more likely to respond after HD-ASCT than others. The question becomes even more relevant considering that in our study, patients who responded to HD-ASCT and especially patients with subjective response within one year after HD-ASCT had significantly longer OS (Fig. 3a). Overall and subjective response also proved to be the strongest independent factors influencing OS in multivariate analysis. In our setting it could be seen that clinical response was associated with less SSc organ involvement. This emphasizes the fact, that close monitoring for organ involvement should be performed and HD-ASCT should not be delayed when organ functions are deteriorating. Furthermore, it highlights the importance of patients´ subjective response, which is not easily quantified. A fact that should be addressed in future prospective trials.

In the last years two different conditioning regimens have been used: The standard CyATG protocol was extended by thiotepa (Henes et al. 2014) or fludarabine (Burt et al. 2021) in order to reduce cyclophosphamide dosage and thereby cardiac and other toxicities. Evidence regarding the TrCyATG protocol is based on a single prospective study comparing survival data from a small number of patients using this protocol with the results of other studies using the CyATG protocol (Giorgetti et al. 2004; Komatsuda et al. 2006; Henes et al. 2014). Henes et al. showed that patients treated with thiotepa needed longer time for hematologic recovery (Henes et al. 2014). This can be confirmed by our study since patients with a thiotepa-based conditioning protocol also had longer time for neutrophil and platelet engraftment. It can therefore be assumed that if TRM of cardiac etiology decreases, an increase in infectious complications must be accepted.

The retrospective design of our study may be considered for limited interpretation of our results. Nevertheless, with this real-world data, important information about patients excluded in other studies due to severe comorbidities or advanced disease stages has been gathered.

In summary, with this study we demonstrated the success and potent therapeutic potential of HD-ASCT in SSc patients. TRM remains a challenge that can be reduced to some degree by eliminating high-risk factors and could be even less, if patients´ organ functions were monitored more closely, in order to avoid missing the right timepoint for HD-ASCT.

## Data Availability

No datasets were generated or analysed during the current study.
